# The p38 Pathway: From Biology to Cancer Therapy

**DOI:** 10.3390/ijms21061913

**Published:** 2020-03-11

**Authors:** Adrián Martínez-Limón, Manel Joaquin, María Caballero, Francesc Posas, Eulàlia de Nadal

**Affiliations:** 1 Institute for Research in Biomedicine (IRB Barcelona), The Barcelona Institute of Science and Technology, Baldiri Reixac, 10, 08028 Barcelona, Spain; adrian.martinez@irbbarcelona.org (A.M.-L.); manel.joaquin@irbbarcelona.org (M.J.); maria.caballero@irbbarcelona.org (M.C.); 2 Departament de Ciències Experimentals i de la Salut, Universitat Pompeu Fabra (UPF), E-08003 Barcelona, Spain

**Keywords:** p38 MAPK, SAPK, phosphorylation, oncogenicity, tumor suppressor, cancer treatment

## Abstract

The p38 MAPK pathway is well known for its role in transducing stress signals from the environment. Many key players and regulatory mechanisms of this signaling cascade have been described to some extent. Nevertheless, p38 participates in a broad range of cellular activities, for many of which detailed molecular pictures are still lacking. Originally described as a tumor-suppressor kinase for its inhibitory role in RAS-dependent transformation, p38 can also function as a tumor promoter, as demonstrated by extensive experimental data. This finding has prompted the development of specific inhibitors that have been used in clinical trials to treat several human malignancies, although without much success to date. However, elucidating critical aspects of p38 biology, such as isoform-specific functions or its apparent dual nature during tumorigenesis, might open up new possibilities for therapy with unexpected potential. In this review, we provide an extensive description of the main biological functions of p38 and focus on recent studies that have addressed its role in cancer. Furthermore, we provide an updated overview of therapeutic strategies targeting p38 in cancer and promising alternatives currently being explored.

## 1. Introduction

Mitogen-activated protein kinase (MAPK) cascades are signaling components that show a high degree of conservation throughout evolution and play a key role in converting extracellular stimuli into a broad range of cellular responses. All MAPK signaling cascades consists of a three-tiered module of protein kinases: MAPK kinase kinases (also known as MKKKs or MAP3Ks) at the top, MAPK kinases (also known as MKKs, MEKs, or MAP2Ks) in the middle, and MAPKs at the bottom [1].

In mammals, three major MAPK cascades have been described. The ERK1/2 pathway is activated mainly by mitogens and has been shown to be upregulated in many human tumors. In contrast, the Jun N-terminal kinase (JNK) and p38 pathways are activated mostly by environmental and genotoxic stresses and are therefore, also generically known as stress-activated protein kinases or SAPKs. The JNK and p38 signaling pathways show a certain degree of redundancy in their actions; however, the extent of crosstalk between them and their implications in cell physiology regulation depends on the cellular type, tissue, and organism. This review outlines current understanding of p38 MAPK family members and their involvement in tumor development, as well as the strategies to treat cancer based on targeting p38.

## 2. p38 MAPK Diversity

The four p38 MAPKs are encoded by distinct genes: p38α (MAPK14), which comprises two different spliced variants; p38β (MAPK11); p38γ (MAPK12); and p38δ (MAPK13) [2,3]. p38α is expressed abundantly in most cell types and thus most of the published literature refers to this isoform. Although ubiquitous, p38β is expressed at very low levels compared to p38α, and its function seems to be redundant with p38α (e.g., [4]). In contrast to these two isoforms, the expression patterns of p38γ and p38δ are more restricted, and these MAPKs may have more specialized functions [5,6].

Several mouse models genetically targeting p38 have been produced. Of note, p38β, p38γ and p38δ knockout mice do not have developmental defects. Moreover, of these three models, only p38δ knockout mice display a clear phenotype. In this regard, these animals are protected against high-fat diet-induced insulin resistance through the regulation of PDK1 activity and insulin secretion. This observation, therefore, indicates that this MAPK isoform plays a key role in the regulation of glucose homeostasis [7,8]. Moreover, p38δ knockout mice are resistant to the development of TPA (12-O-Tetradecanoylphorbol-13-acetate)-induced skin papilloma [9], which is in agreement with reports showing the relevance of this isoform in keratinocyte cell differentiation and survival [10]. In contrast, p38α knockout mice die at the embryonic stage due to placental morphological defects [11,12], a phenotype that resembles the MKK3 and MKK6 double knockout. This indicates that these two MAP2Ks are genetically epistatic to p38 [13].

## 3. p38 MAPK Activation

Full activation of most protein kinases requires phosphorylation on a flexible motif known as the activation loop. In particular, p38 activation takes place by the dual phosphorylation of Thr and Tyr on the Thr‒Gly‒Tyr motif located on the activation loop found on the kinase subdomain VIII [14]. Dual phosphorylation at these two particular sites alters the folding of p38 by stabilizing the activation loop in a more open conformation and causing rotation between the two major lobules, which allows for substrate recognition and increases the activity of the kinase. The MAP2Ks most likely responsible for phosphorylating p38 in vivo are MKK3 and MKK6 [15,16,17]. Indeed, MKK-targeted gene disruption and siRNA approaches have shown that these MAP2Ks convey the signaling in response to most stress stimuli [18]. Exceptionally, ultraviolet radiation has also been shown to activate p38 through JNK activators such as MKK4 [13,14]. Activation of MKK3 and MKK6 occurs upon phosphorylation of two conserved serine (Ser) and threonine (Thr) residues on their activation loop by a broad range of MAP3Ks, including ASK1 (apoptosis signal-regulating kinase 1), DLK1 (dual-leucine-zipper-bearing kinase 1), TAK1 (transforming growth factor β-activated kinase 1), TAO (thousand-and-one amino acid) 1 and 2, TPL2 (tumor progression loci 2), MLK3 (mixed-lineage kinase 3), MEKK (MAPK/ERK kinase kinase) 3 and MEKK4, and ZAK1 (leucine zipper and sterile-α motif kinase 1). Consequently, the signaling events taking place in MAP3K are rather complex. Indeed, the diversity of MAP3Ks and their regulation provide the cells with a plethora of mechanisms capable of responding to diverse stimuli [19].

**Figure 1:**
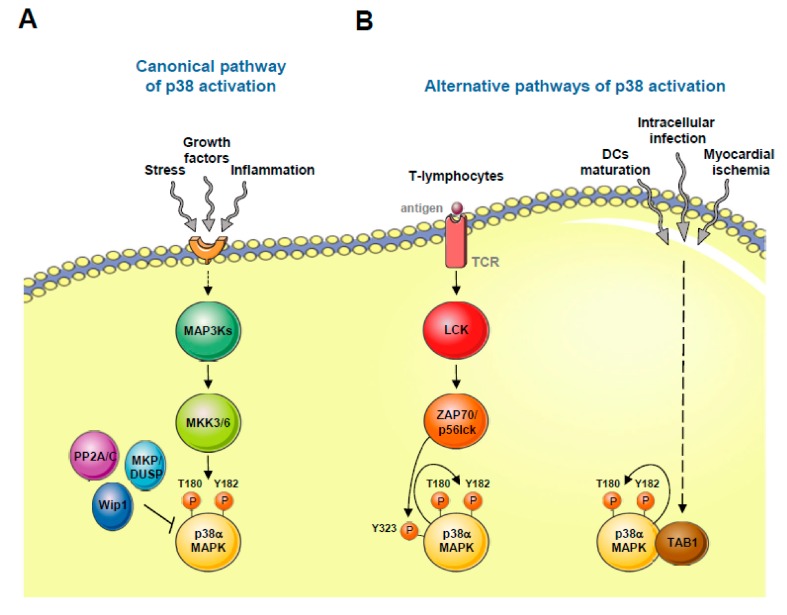
p38 MAPK activation. (**A**) Canonical pathway: Several environmental stimuli activate p38 by phosphorylation of Thr180 and Tyr182 through MAP3Ks and MAP2Ks MKK3/6. Phosphatases PP2A/C, Wip1 and MKP/DUSP inhibit p38 activation. (**B**) Alternative pathways: Noncanonical p38 activation occurs in T-lymphocytes upon antigen presentation (TCR: antigen T cell receptor) and involves phosphorylation of Tyr323, which promotes an auto-phosphorylation loop. In addition, p38 can be activated by the presence of other stimuli such as intracellular infection, myocardial ischemia or dendritic cells (DCs) maturation signals. In these cases, TAB1 associates with p38, promoting its auto-phosphorylation. This figure was created using Servier Medical Art templates, which are licensed under a Creative Commons Attribution 3.0 Unported License; https://smart.servier.com.

The p38α isoform can be activated by noncanonical mechanisms (see Figure 1). One of these alternative mechanisms is restricted to Th1 lymphocytes. In this regard, upon antigen T cell receptor (TCR) stimulation, p38α becomes phosphorylated at Tyr 323 through the action of the kinases ZAP70 (ζ -chain associated protein kinase of 70 kDa) and p56lck. Tyr 323 phosphorylation promotes the capacity of p38α to autophosphorylate, thereby activating itself [20]. The biological relevance of this mechanism of p38α activation has been addressed in p38α-knockin mice, in which Tyr 323 is replaced by the non-phosphorylatable phenylalanine. These mice are viable and fertile but show a defect in p38α activation upon TCR stimulation and in IFNγ (interferon γ) production [21]. Alternatively, p38α can be activated by binding to the protein TAB1 (TAK1-binding protein 1), which again results in p38α autophosphorylation and consequent kinase activation [22,23]. Although this mechanism is not easy to recapitulate with in vitro purified proteins [24,25] it appears to contribute to p38α signaling in intracellular infection, myocardial ischemia, or dendritic cells (DCs) maturation signals [25,26,27,28,29]. Finally, a third noncanonical and MAP2K-independent mechanism leading to p38 signaling has been shown in tumor cells depleted of the DNA replication initiation factor Cdc7. These cells undergo programmed cell death in an ATR- and p38-dependent manner, thereby suggesting that p38 mediates apoptosis upon replication stress [30].

## 4. Substrate Recognition and p38 MAPK Signaling Downregulation

The strength and dynamics of p38 pathway signaling are key elements in determining proper cellular responses [31]. Moreover, population signaling dynamics are qualitatively different from single-cell behaviors. Recently, live cell imaging using KTR (Kinase Translocation Reporter) technology has allowed to dynamically measured JNK, p38, and ERK MAPK activities simultaneously in single living cells [32]. p38 can be activated by a broad range of stimuli, which may engage various signaling elements and substrates, resulting in distinct cellular outcomes. Indeed, strong and sustained p38 activation has been linked to apoptosis, senescence, and terminal cell differentiation, whereas low p38 activation has a cell survival effect [33,34]. A quantitative comprehensive understanding of these signaling decisions has not been achieved to date, but it is likely to identify the mechanisms by which p38 sometimes exerts opposing effects.

To date, a full characterization of the p38 phosphoproteome has not been performed [35]. All p38 substrates identified so far are phosphorylated at Ser or Thr residues usually followed by a proline (Pro). Of note, this Ser/Thr‒Pro motif is particularly abundant in most mammalian proteins, thus specific mechanisms may operate to ensure proper MAPK substrate recognition and signaling exclusivity. Kinase‒substrate recognition relies on the binding between the catalytic cleft of the kinase and the substrate phospho-acceptor residue. On top of that, several p38 substrates harbor a docking site region known as the D domain, which consists of several basic residues that interact with specific acidic residues of p38 [36,37,38]. However, this mechanism of substrate recognition does not account for all the targets described for p38. In many cases, substrate specificity depends on other factors, such as substrate concentration, availability, interacting partners or subcellular localization.

Upon phosphorylation-mediated activation, p38 can in turn phosphorylate both cytoplasmic and nuclear proteins. In order to have access to nuclear substrates, activated p38 transiently accumulates and translocates to the cell nucleus. Since MAPKs do not have nuclear localization or nuclear export motifs, the molecular mechanism by which translocate into the nucleus is not fully known. Given that p38α can genetically complement a budding yeast strain lacking the p38-homologous Hog1 MAPK [39], initial studies on MAPK nuclear shuttling were performed in this single cell organism. Phosphorylation at the Thr‒Gly‒Tyr activation motif is a mark that allows Hog1 to rapidly translocate into the nuclear compartment, where it gains access to its nuclear substrates. Later on, phosphatases inactivate the kinase, allowing its accumulation to the cytoplasm [40,41]. In mammalian cells, the nuclear translocation of p38 also requires phosphorylation of the Thr‒Gly‒Tyr activation motif. The activated MAPK is then transported by binding with β-like importins and transport is mediated by microtubules and dyneins [42,43]. Moreover, p38 nuclear export requires kinase dephosphorylation and takes place in association with MK2, a known downstream substrate of p38 [44].

Finally, p38 signaling ends upon the removal of the phosphates from the Thr‒Gly‒Tyr motif. Several Ser/Thr phosphatases, such as PP2A and PP2C, have been described to remove the threonine phosphate on the activation motif. The action of these phosphatases leads to the appearance of Tyr monophosphorylated p38 forms that lack kinase activity [45]. In addition, several phosphatases are upregulated at a transcriptional level upon stress, thereby contributing to the termination of p38 stress-mediated signaling. The Ser/Thr phosphatase Wip1 is upregulated by p53 when cells are exposed to ultraviolet light, and this upregulation contributes to the downregulation of p38 signaling, thereby allowing damaged cells to recover [46]. However, Wip1 has also been described to dephosphorylate other DNA damage response proteins, indicating that it might promote a p38-independent regulatory effect [47]. Stress-activated p38 also contributes to the gene expression of several members of the DUSP/MKP (dual specificity phosphatases/MAPK phosphatases) family, the most relevant being Dusp1/MKP1 [48]. DUSPs are capable of dephosphorylating both residues of the Thr‒Gly‒Tyr activation motif, and some members of this family have docking domains to interact with p38 [49].

## 5. p38 MAPK Activators and Physiological and Cellular Functions

p38 is activated by a wide range of environmental stimuli (e.g., heat shock, changes in osmolarity, and oxidative stress), genotoxic and DNA damaging agents (e.g., cisplatin, doxorubicin, ultraviolet light, and γ-radiation), inflammatory cytokines, PAMPs (pathogen-associated molecular patterns), and DAMPs (danger-associated molecular patterns) [6].

p38 is a multitasking kinase that regulates multiple cellular functions, including cell proliferation, differentiation, stress response, apoptosis, and cell migration and survival, among others, by interacting with a plethora of substrates [3,50]. Part of this regulation is the result of strong transcriptional regulatory activity. p38 activation leads to the phosphorylation of many transcriptional regulators that coordinate particular gene expression programs. p38-dependent transcriptional response under stress conditions has been observed to be time-dependent and stimulus-specific [48], thereby indicating that p38 interacts with additional pathways and in combination with non-p38 regulated transcription factors to elicit distinct responses.

The contributions to physiology of p38 have been addressed, mostly by using inhibitors that block kinase activation, as well as by using genetically engineered mouse models. Since mice lacking p38α die before birth, some of the strategies to overcome this include the generation of p38α tissue-specific conditional knockouts, deletion of upstream or downstream proteins of the MAPK pathway like MKK3/6, MSK1/2, MK2, or WHIP1, and generation of dominant negative p38α forms [51].

The ablation of p38α leads to an increased proliferation of hematopoietic progenitor cells through the regulation of Epo expression [52], as well as primary cardiomyocytes through the downregulation of mitotic genes such as Cyclin A and Cyclin B [53]. Genetically modified mice carrying a kinase-dead allele of p38α, where the Thr‒Gly‒Tyr activation motif has been changed to Ala‒Gly‒Phe, display hyperproliferation of pancreatic islets due to reduced expression of CDK inhibitors [54]. All together, these data show that p38α limits the proliferation of several cell types and that it may therefore have a putative tumor suppressor function. Indeed, mice lacking p38α are susceptible to developing K-RasG12V-induced lung tumors [55] and chemically induced liver cancer [56,57]. The role of p38 in skeletal muscle differentiation has been known for many years. Constitutive activation of p38 signaling regulates the myogenic program by specifically controlling skeletal muscle gene expression and the recruitment of p38 and chromatin remodelers to specific skeletal gene promoters [58,59,60]. The study of genetically modified mice shows that p38α ablation has an important role in myoblast differentiation and myotube formation [61], whereas other forms of the kinase such as p38γ may contribute to regulating the expansion of satellite cells [62]. The observation that p38 signaling is activated by PAMPs indicates that this cascade participates in the innate immune response.

## 6. Regulation of Cell Cycle by p38 MAPK

Of particular importance is the capacity of p38 to control cell cycle progression, which is crucial for cellular homeostasis. p38 regulates proliferation at both G1/S and G2/M phases of the cell cycle by activating checkpoint responses [63].

Exposure to osmotic stress, reactive oxygen species, DNA damage, and prosenescence stimuli activates p38 signaling, thereby leading to the induction of the G1/S checkpoint. p38 activity promotes the downregulation of cyclin D1, which is essential for S phase transition. This downregulation is achieved by stimulating ubiquitin-dependent degradation of cyclin D1 [64] and inhibiting cyclin D1 gene transcription through activation of HBP1 [65]. Additionally, p38 regulates p53 activation either directly or indirectly through HBP1 [66]. p53 acts as a tumor suppressor by controlling the G1/S checkpoint, and its activation results in the upregulation of p21Cip1/WAF1, GADD45, and 14-3-3σ proteins, hence inducing proliferation arrest [67,68]. Furthermore, p38 also induces G1/S cell cycle arrest by directly or indirectly activating the CDK inhibitors p21Cip1 [69], p27Kip1 [70], and p57Kip2 [71]. Remarkably, p38 also inhibits the E2F transcription factor, which regulates the expression of the retinoblastoma protein (RB) [72]. It has been shown that p38 phosphorylates the N-terminal region of RB, rendering it insensitive to CDK/Cyclin-mediated inactivation and delaying cell-cycle progression [73]. p38 also controls G2/M phase transition. This regulatory mechanism has been described mostly in the context of the cellular response to DNA damage. Both ATM/ATR Ser/Thr protein kinases can sense DNA damage and activate p38 via MKK3/6 [74]. Furthermore, p38-dependent p53 activation promotes the transcription of GADD45α, which binds to the Cdk1/cyclin B complex to delay G2/M phase [75]. In fact, in p53-deficient cells, p38 inhibits Cdc25B phosphatase activity through MK2 phosphorylation, resulting in cell cycle arrest in G2/M [76,77].

Notably, and contrary to what is commonly reported, p38 can enhance proliferation. However, this effect has been observed only in hematopoietic cells and in some cancer cell lines [78]. Such an apparently contradictory role in the regulation of the cell cycle has been explored in depth [79]. Phosphorylation of p38 occurs both under mitogenic stimulation and in the presence of an acute cellular stress, and differences in the duration and intensity of such activation modulate the pathway to induce either proliferation or cell cycle arrest. However, the molecular mechanisms and biological contexts in which p38 activation favors cell division remain largely unexplored.

## 7. p38 MAPK as a Tumor Suppressor

**Figure 2:**
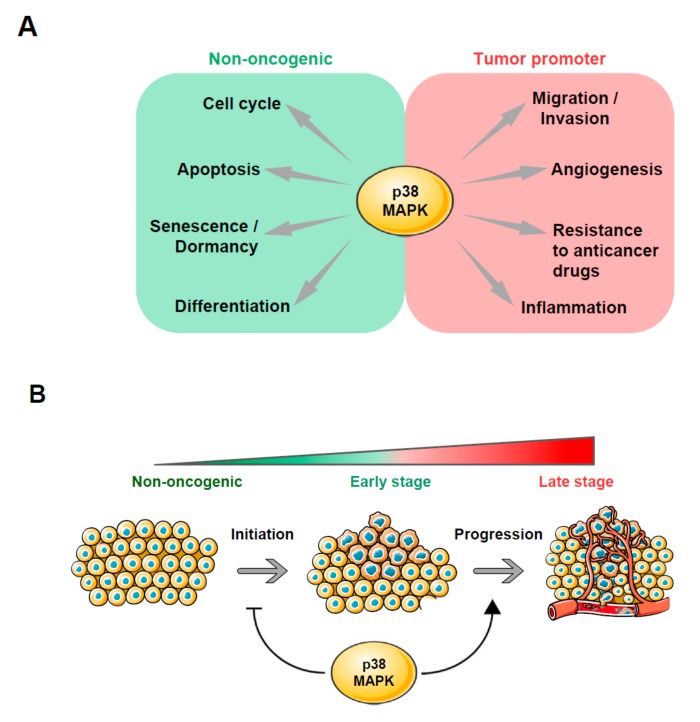
The dual role of p38 MAPK in tumorigenesis. (**A**) p38-dependent cellular. processes can be classified on the basis of the role they play during tumorigenesis. (**B**) p38 inhibits oncogenic transformation and promotes the progression of tumors in late stages of the disease. This figure was created using Servier Medical Art templates, which are licensed under a Creative Commons Attribution 3.0 Unported License; https://smart.servier.com.

The role of p38 in cancer has been extensively studied. Several reports have shown that p38 functions as an antitumorigenic factor (see Figure 2). Its role during adaptation to stress, and especially in promoting cell cycle arrest and differentiation, supports the notion that the stress-activated kinase is a tumor suppressor. Initial studies showed that p38 activation inhibits Ras-induced transformation through cyclin D1 downregulation. p38α is phosphorylated under Ras hyperactivation and exerts a negative regulatory feedback loop by inhibiting Ras-dependent gene expression and cell growth [80]. Other publications have also reported that p38α acts as a ROS-sensing agent in mutant Ras-induced transformed cells. Exacerbated ROS production occurs under oncogenic Ras expression and is known to enhance tumorigenesis. p38 activation triggers apoptosis upon bursts of oxidative stress in these cells [81]. Experiments targeting members of the p38 pathway other than p38 have led to similar conclusions. For example, mice lacking MKK3 and MKK6 show a higher tendency to develop tumors [78]. Also, expression of MKK3 was found to be significantly diminished in five out of eight distinct cancers analyzed and restoration of the kinase activity inhibited cell growth in vitro by stimulating CDK inhibitor proteins p21 and p27 [82]. The downregulation of cyclin expression mediated by p38-phosphorylated RB also prevents cell proliferation in several cancer cell lines [72]. Remarkably, induction of TGFβRII in metastatic prostate cancer cells mediates RB phosphorylation by p38, which prevents bone metastasis [83]. On the other hand, inactivation or depletion of the PPM1D (or WIP) phosphatase with resultant p38 activation suppresses mammary tumorigenesis through p38-mediated activation of the p16 pathway [84]. Similarly, other negative regulators of p38, such as the DUSP26 phosphatase and PPMID, have been found to be overexpressed in breast and thyroid cancers [85,86].

The importance of the p38 pathway in the regulation of the immune response has attracted considerable attention in the context of carcinogenesis. Immune cells can strongly modulate tumor progression by secreting cytokines and chemokines. Indeed, cancer and inflammation are tightly associated, and some chronic inflammatory diseases, particularly those occurring in the gastrointestinal tract, correlate with a higher risk of cancer development [87]. p38 regulates the production of tumor necrosis factor (TNF), interleukin 6 (IL-6) and other cytokines like IL-1, COX2, and IL17, which regulate growth and survival and therefore show remarkable protumorigenic capacities. The details of the relationship between tumor development and the p38-dependent immune response have not been fully characterized. For instance, inflammation-associated development of hepatocellular carcinoma is enhanced by p38α loss in a mouse model [56], thereby suggesting that this kinase participates in modulating inflammation-dependent transformation [51]. In contrast, some reports coincide on the pro-inflammatory and protumorigenic roles of p38 [88,89].

The role of p38 in promoting cell differentiation has also been reported to be relevant for its tumor inhibitory activity. In rhabdomyosarcoma cells, expression of either MKK3 or MKK6 reactivates the pathway, leading to terminal differentiation of cancer cells [33]. Deletion of p38α in mice increases proliferation and impairs the differentiation of lung stem and progenitor cells, thereby exerting an overall negative effect on tissue homeostasis. The protein levels of both C/EBPα and HNF3β, two well-known transcription factors involved in lung differentiation, were found to be reduced in the lungs of p38α-deficient mice. In contrast, epidermal growth factor receptor (EGFR) and AKT transcription increased, which explains the exacerbated proliferation of these tumor cells [55]. Interestingly, the same p38α-deficient mice that overexpressed mutant K-Ras showed larger tumors than those in animals harboring a wild-type kinase. This finding indicates that modulation of differentiation signals in the lung by the kinase has an impact on tumor formation [55]. Although it is known that p38 activity also promotes cellular differentiation in other tissues, our understanding of how pathway-dependent differentiation cues affect tumorigenesis is still very limited.

Additionally, the pro-apoptotic activity of p38 is related to its ability to inhibit tumor growth. This is particularly evident in cases where the effects of chemotherapeutic drugs rely on p38 activation [90]. Another process that counteracts carcinogenesis is senescence. Cells that undergo senescence can be classified based on several biological features, although the most notorious one is the permanent blocking of cell division. The anti-oncogenic role of p38 might derive from its capacity to force senescence in certain contexts. In support of this notion, hyperactivation of Ras in primary cells resulted in premature senescence as a result of p38 and HBP1 activation [65]. Finally, dormancy induction in tumor cells is regulated by p38 activity through its downstream effector MSK1. In patients with ER+ breast cancer, low MSK1 expression associates with early metastasis. MSK1 downregulation impairs the differentiation of breast cancer cells, increasing their bone homing and growth capacities [91]. TGFβ2 stimulation reduces ERK1/2 and increases p38 activity, thereby suppressing metastasis in human head and neck squamous cell carcinoma (HNSCC) cells. In these cells, p38 inhibition induces tumor proliferation [92], implying that molecular switching between proliferative and dormant states in tumors might be dependent on this kinase.

## 8. p38 MAPK as a Tumor Promoter

Despite numerous studies that provide experimental evidence of the antitumorigenic role of p38, many others demonstrate that this kinase promotes cancer by enhancing survival, migration, or resistance to stress and chemotherapeutic agents in tumor cells [78] (see Figure 2). p38α was found to be required for tumor progression in a mouse model of breast cancer, where p38α deletion impaired the DNA damage response and increased replicative stress, DNA damage and chromosome instability (CIN) [93]. Also in a model of breast cancer, p38δ deletion reduces tumor volume [94]. Consistent with its tumor promoter role, p38 kinase activation was found to be higher in a panel of 18 lung-resected tumors when compared to normal tissue samples [95]. Similarly, immunohistochemical analysis of a massive collection of more than 400 human HNSCC tissue samples showed that p38 was hyperactivated in 79% of cases [96].

In contrast to the p38α tumor-suppressing role, p38γ is required for Ras transformation in a phosphorylation-independent manner. In this regard, the expression of p38γ is selectively induced in colon cancer cells harboring mutated Ras and is required for proliferation [80,97], thereby indicating that isoform-dependent mechanisms act on Ras signaling. Moreover, p38γ is essential in liver tumorigenesis; in mouse hepatocytes, p38γ induces proliferation after partial hepatectomy by promoting the phosphorylation of retinoblastoma (RB) tumor suppressor protein at known CDK target residues. Indeed, lack of p38γ or treatment with the p38γ inhibitor protects against the chemically induced formation of liver tumors [98]. Such isoform specificity is also observed in other contexts. For instance, AP-1-dependent transcription and cell proliferation in MCF-7 breast cancer cells are positively regulated by p38β but inhibited by p38δ and p38γ [99].

Compared to other genes that present recurrent mutations in several cancer types, the p38 gene does not show systematic loss-of-function mutations in common human malignancies. In total, results obtained from the sequencing analysis of a pool of 1500 samples revealed that the frequency of p38 mutations (counting all four isoforms) in human cancers is lower than 1% [100]. This incidence could be related to the pleiotropic nature of this signaling pathway and its capacity to regulate multiple cellular functions in diverse contexts. In fact, the functional contribution of the pathway to the pathology most likely depends on a range of factors, such as cell type, kinase expression levels, and crosstalk with other pathways. Therefore, cancer cells might feed from the high versatility of the pathway, and thus similar mutations are not likely to accumulate in different type of tumors [78].

The stage of tumor development could be a strong component governing the function of p38 in tumorigenesis. Cancer is a highly complex pathology in which genetic and metabolic rearrangements occur in every step. Therefore, tumor requirements are completely stage-dependent. In general, experimental evidence indicates that low p38 activity impairs tumor formation and growth during early stages of the disease, while more advanced tumor stages can benefit from higher activation of the pathway [90]. An extensive immunohistochemical staining analysis of human prostate tissue samples was performed to explore this apparent contradiction. Interestingly, the expression levels of the phosphatase DUSP1 were found to be extremely high in early stages of cancer, whereas they decreased markedly as the malignancy progressed [101]. The dual role of p38 in tumorigenesis was also explored by Gupta et al. (2014), who showed that p38α suppresses inflammation-associated intestinal epithelial damage and tumorigenesis but contributes to the proliferation and survival of colon tumor cells [102]. New studies are required to determine whether this duality is recapitulated in other malignancies.

Cancer is not simply a collection of cells that develop independently from their surroundings. The relevance of the tumor microenvironment as an active component of cancer development has gained special attention in recent years, although it is still a largely unexplored field. Extensive communication between cancerous and nontransformed cells takes place through a continuous flux of cytokines, chemokines, growth factors, and extracellular enzymes and determines tumor survival and progression [103]. A recent study reported the effect of co-culturing primary cancer-associated fibroblasts (CAFs) with ovarian cancer cells. The presence of CAFs boosted tumor proliferation and metastasis by promoting changes in cancer cell metabolism that support the invasive phenotype. Of note, p38α activation in CAFs is necessary to orchestrate this crosstalk [104]. Inflammatory responses mediated by p38 are also expected to modulate the tumor microenvironment in other contexts. In this scenario, two distinct perspectives are needed: one that focuses on the tumor itself and the other that evaluates the contribution of p38 signaling in stromal cells. New lines of research are required to elucidate such regulatory mechanisms.

Tumor cells can detach from the initial tumor mass to migrate and invade other tissues, severely affecting homeostasis. This invasive process, known as metastasis, is by far the highest risk factor in cancer and is estimated to account for about 90% of deaths in cancer patients [105]. There is a growing evidence about the pro-oncogenic role of p38 during late stages of tumorigenesis involving migration to neighboring organs. High expression of matrix metalloproteinases (MMPs) in human cancers strongly correlates with metastasis, and therefore with reduced survival rates [106]. MMPs are either secreted or associated to the plasma membrane and are responsible for the degradation of components from the extracellular matrix (ECM), such as collagen, fibronectin, and plasminogen. Some studies have shown that p38α regulates the expression of several MMPs, which suggests a close relationship between p38 activation and metastasis. Indeed, inhibition of p38 diminishes MMP-2/9 activities and the invasive capacity of bladder cancer cells in vitro [107]. Moreover, p38 inhibition prevents TGF-β-dependent MMP-9 expression and reduces bone metastasis from breast cancer in mice [108] and, as mentioned before, also blocks bone metastasis from prostate cells [83].

p38 might also be involved in tumor metastasis via other mechanisms. For instance, p38 reduces the expression of the extracellular glycoprotein fibulin 3, which regulates cell migration [109]. Indeed, inhibition of p38 activity blocks HeLa cell migration induced by chemo-attractants such as CXCL12 or complement factor 5a (C5a) [110]. Moreover, p38α induces the expression of vascular endothelial growth factor (VEGFA) and hypoxia inducible factor 1α (HIF1α), which are both strong pro-angiogenic molecules and therefore promote tumor vascularization and metastasis [78]. Likewise, p38 modulates the expression of pro-angiogenic factors such as HBEGF, IL-8 and VEGFA in metastatic breast carcinoma cells. Inhibition of stress-activated p38 reduces primary tumor growth in orthotopic xenograft models and the number of lung metastatic colonies after tail-vein injection [111]. Although these findings support the prometastatic role of the p38 pathway, it has been reported that p38-knockdown human colorectal cancer cells show a greater capacity to colonize the lung in an orthotropic xenograft mouse model [112]. This observation would suggest that, in some cases, p38 signaling might inhibit metastasis.

## 9. Targeting p38 MAPK for Cancer Therapy

As stated above, abundant experimental evidence shows that p38 can exert pro-oncogenic functions in various types of cancer. This finding opens up the possibility of developing cancer therapies based on small inhibitory compounds. Seven clinical trials targeting p38 for the treatment of cancer are currently underway (see Table 1). One example is Ralimetinib (or LY2228820), which is a potent and selective inhibitor of p38α and p38β. Ralimetinib is now being tested, both alone and in combination with other agents, for the treatment of ovarian cancer, glioblastoma, and metastatic breast cancer [113]. Notably, some p38 inhibitors are also being tested for the treatment of other pathologies, such as rheumatoid arthritis and Parkinson’s disease [114].

Although many efforts have been channeled into developing effective p38 inhibitors for clinical practice, almost all the trials performed to date in patients have failed, and no compounds have yet been approved. The appearance of systemic side effects in the heart, liver, and nervous system caused by such small molecule inhibitors accounts for the failure of many of these trials [115]. These systemic side effects could be attributed to the multifunctional character of p38 and the fact that it regulates more than 60 proteins (at least 66 p38 substrates have been described to date) [35]. Although in vivo experiments show that p38 inhibitors can suppress tumor progression in many cases, it remains possible that the inhibition of this kinase would favor tumor initiation in other tissues subjected to oncogenic stimuli. Recent results could offer a solution to overcome these difficulties. According to several studies, p38 is involved in the response to DNA damage and resistance to chemotherapeutic agents [90]. Therefore, one approach that is gaining relevance relies on the synergistic effect observed from combining classical anticancer drugs and p38 inhibitors. For instance, patient-derived breast tumor xenografts treated with p38α inhibitors show higher sensitivity to treatment with taxanes [116]. A similar result was also observed using cisplatin in breast cancer cells [117]. Also, p38 inhibition decreases MDR1 levels and sensitizes human gastric cancer cells to epirubicin, 5-fluoroacyl, and cisplatin [116]. Other tumor cells also appear to benefit from the combined therapy with chemotherapeutic drugs, thereby indicating that this effect can be recapitulated in several contexts, thus underscoring the potential for treatment. In this regard, a number of clinical studies are currently testing the effects of these combination therapies in clinics. As mentioned above, promising results have been obtained in trials with the p38α inhibitor Ralimetinib in combination with radiotherapy and Temozolomide for the treatment of patients with glioblastoma, as well as with Gemcitabine to treat patients with ovarian cancer [113].

A second approach to improve the effectiveness of cancer treatments involves targeting other kinases downstream the p38 pathway, thus limiting the response to only a subset of p38-mediated responses. For instance, the modulation of MAPKAPK2 (MK2), which is a direct downstream kinase of p38 that regulates several processes, including apoptosis, cell cycle, and stress response to oxidizing agents. MK2 regulates the expression of pro-inflammatory factors and responds to DNA damage by inducing the G2/M checkpoint response, thereby contributing to tumor growth and invasiveness in several cancer types [115,118,119]. Notably, MK2-deficient mice are refractory to skin tumor development as a consequence of a reduced pro-inflammatory response and increased apoptosis [120]. A recent study partially recapitulated these results using a model of colitis-associated carcinogenesis, but without involving an inflammatory response [121]. Similar to p38, MK2 could be a good target in combination with other drugs. Inhibition of MK2 causes the regression of p53-deficient tumors in vivo after treating mice with Cisplatin and Doxorubicin [76]. Furthermore, MK2 inhibition in combination with IAP (inhibitor of apoptosis proteins) inhibitors kills acute myeloid leukemia (AML) cells [122]. These examples demonstrate that MK2 inhibitors can be used alone or synergistically with other drugs like DNA-damaging agents for cancer therapy.

The retinoblastoma (RB) protein emerges as another interesting potential candidate and a therapeutic target downstream of p38. RB is essential for the proper modulation of the G1/S cell cycle transition, and its inactivation contributes to deregulated cell proliferation, which is a hallmark of cancer, thus leading to tumorigenesis [123]. As mentioned above, N-terminal RB phosphorylation on Ser249/T252 sites by p38 is dominant over the effect of CDK-induced phosphorylation, which is a hallmark of many types of cancer. The phosphomimetic RB^S249E/T252E^ mutant acts as a super-repressor and blocks cyclin expression, preventing cell proliferation in all the cancer cell lines tested so far (i.e., bladder, pancreas, prostate, and breast) and leading to reduced tumor formation in a mouse xenograft model of triple-negative breast cancer (TNBC) [72,73,83]. Furthermore, RB phosphorylation on Ser249/T252 promotes tumor immunity by inhibiting NF-κB transcriptional activity and PD-L1 expression. Indeed, the expression of an RB-derived S249/T252 phosphomimetic peptide suppresses radiotherapy-induced upregulation of PD-L1 and increases the therapeutic efficacy of radiation in vivo [124]. In this scenario, the increased tumor suppressor capacity of RB mediated by the phosphorylation of Ser249/T252 opens up the possibility of developing a new generation of cancer drugs that mimic the effects observed by these phosphorylation events, thereby increasing the repressor capacity of RB. Many human tumors with high CDK-Cyclin levels still carry a functional RB gene [123], so patients with such tumors may be treated with these compounds.

## 10. Concluding Remarks

Despite the limited success of medicinal chemistry to design a drug that targets p38 with a clear therapeutic effect, this stress-activated kinase or its downstream components remain an attractive candidate for the development of new therapies. Indeed, there is still very limited knowledge about some basic aspects of the functionality of the p38 pathway. For instance, p38 isoform-specific biological functions remain a mystery. Specifically, p38δ and p38γ, which have unique expression patterns and perform a different set of functions, have received less attention than p38α and p38β.

p38 has classically been considered a tumor suppressor gene, in spite of the lack of mutations in the genomic sequence in human cancers. However, there is now a large collection of publications supporting a tumor promoter role for p38 and thus in disagreement with its former classification as a prominently anticancer protein. In particular, the role of p38 in metastasis is gathering attention exponentially, since advanced tumorigenic stages still represent a major health challenge worldwide. Nevertheless, even with the increasing body of evidence and studies available, a clear picture of the contribution of p38 to tumorigenesis is still missing. In this regard, many cancer-related studies lack an isoform-dependent approach and hence their translational potential is limited. In this regard, the way each p38 isoform, especially δ and γ, contributes to oncogenesis is not well understood and the results can often be controversial. Moreover, the large versatility of the stress-activated pathway is probably another important handicap to the effectiveness of therapies. It is evident that the factors mentioned above have hindered the development of more effective and safer drugs for specific malignancies.

A deeper understanding of the p38 pathway will translate into better therapeutic strategies for the treatment of cancer, with overall higher success rates. Nevertheless, and considering what has been explained above, it should be taken into account that, while p38 inhibition-based therapies efficiently block tumor growth, they might simultaneously facilitate malignant initiation in other tissues subjected to oncogenic stimuli. As discussed in the previous section, other therapeutic options are also being explored intensively, with the aim of overcoming this hurdle. One particular alternative involves the combination of existing drugs with p38 small inhibitors, while another seeks to target upstream or downstream regulatory factors within the pathway. The rationale behind the latter is that a focus on other regulatory proteins might bypass the undesirable pleiotropic effects caused by p38 inhibition. In particular, recent studies have been devoted to MK2 kinase downstream of p38, and to RB, a p38-dependent transcriptional regulator of cell proliferation. Remarkably, experiments targeting these two proteins have shown promising results in vivo, thereby reflecting their exceptional potential for clinical applications.

## Figures and Tables

**Table 1:** LY3007113, LY2228820 (Ralimetinib), and SCIO-469 are p38 MAPK inhibitors. Information was obtained from clinicaltrials.gov.

Cancer Type	Treatment	Phase of Study	Details
Advanced and/or metastatic cancer	LY3007113	I	Evaluation of the safety and tolerability to different doses of treatment
Relapsed ovarian cancer after platinum-based chemotherapy	LY2228820 Carboplatin Gemcitabine	Ib/II	Evaluation of the safety of treatment based on inhibitor plus chemotherapy
Advanced cancer	LY2228820	I	Evaluation of the safety and tolerability to different doses of treatment
Metastatic breast cancer	LY2228820 Tamoxifen	II	Evaluation of the efficacy of inhibitor plus tamoxifen
Adult glioblastoma	LY2228820 Temozolomide (TMZ) Radiotherapy	I/II	Determination of inhibitor dose with TMZ and radiotherapy (phase I). Estimating the six-month Progression-free survival (PFS) rate (phase II)
Relapsed multiple myeloma (MM)	SCIO-469 Bortezomib	II	Evaluation of the efficacy of inhibitor in patients with MM
Myelodisplastic syndrome (MDS)	SCIO-469	II	Evaluation of the safety, tolerability and efficacy of inhibitor in patients with MDS
